# Adverse Materno-Foetal Outcomes of Pre-Eclampsia at a Rural Tertiary Hospital in the Eastern Cape Province of South Africa

**DOI:** 10.3390/ijerph23010016

**Published:** 2025-12-22

**Authors:** Nomvuyiso Nqala, Lizo Godlimpi, Akhona Ncinitwa, Mirabel Kah-Keh Nanjoh

**Affiliations:** Department of Public Health, Faculty of Medicine and Health Sciences, Walter Sisulu University, Mthatha 5099, South Africa; mvuyienqala@gmail.com (N.N.); lizogodlimpi8@gmail.com (L.G.); ancinitwa@wsu.ac.za (A.N.)

**Keywords:** pre-eclampsia, maternal outcomes, foetal outcomes, rural, South Africa

## Abstract

Pre-eclampsia affects several physiological systems, often changing the course of pregnancy and manifesting with both maternal and foetal adversities, with a higher burden in rural Sub-Saharan African settings. This study presents maternal and foetal adverse outcomes associated with pre-eclampsia at a rural tertiary hospital in the Eastern Cape Province of South Africa. A prospective analytical case-control study was conducted with 250 pregnant women planned for delivery at the study setting’s labour unit. Pregnant women with pre-eclampsia were considered cases, whereas pregnant women without pre-eclampsia were considered controls. Cases were enrolled first, followed by a matched pair of controls based on their gravidity. A consecutive sampling technique was used to recruit eligible cases and controls. Data was collected using a self-designed questionnaire followed by descriptive and inferential analysis. Adverse foetal outcomes associated with pre-eclampsia were low birth weight [Adjusted odds ratio (AOR) = 2.1, *p* = 0.006] and foetal distress (AOR = 2.5, *p* < 0.001). Maternal outcomes associated with pre-eclampsia were haemolysis, elevated liver enzymes, and low platelets syndrome (AOR = 42.7, *p* < 0.001), as well as preterm delivery (AOR = 3.0, *p* = 0.001). Early antenatal visits, continuous monitoring of pre-eclamptic pregnant women, and implementation of preventive and curative measures to reduce the possibilities of this condition and its adverse outcomes are needed.

## 1. Introduction

Pre-eclampsia is a pregnancy-related condition that has only been documented in humans and forms one of the bands of the spectrum of hypertensive disorders of pregnancy [1]. In this category of pregnancy disorders, pre-eclampsia stands as a forewarning for eclampsia, while chronic hypertension and gestational hypertension often predispose to the condition [1,2]. Pre-eclampsia usually occurs after 20 weeks [3]; cases with onset before 20 weeks have also been reported [4]. The diagnostic features of pre-eclampsia are hypertension (systolic blood pressure measurement of 140 mmHg and/or diastolic blood pressure measurement of 90 mmHg) alongside proteinuria in a previously normotensive and non-proteiniuric pregnant woman [5]. Demographic, obstetric, clinical, and genetic factors, including older maternal age, obesity, familial history, primigravity, nulliparity, history of pre-eclampsia, persistent hypertension, diabetes, cardiac disease, and renal diseases, have contributed significantly to the development of pre-eclampsia during pregnancy [2,6,7]. Nowadays, pre-eclampsia is one of the clinical issues that obstetricians face and represents a significant public health challenge due to its associated burden of high adverse pregnancy outcomes. Statistics reveal a global prevalence rate of 2% to 8% [8], with increasing prevalence reported from country-specific empirical studies [9,10,11].

The occurrence of pre-eclampsia alters the course of pregnancy and influences several aspects of pregnancy outcomes. It had been connected with maternal mortality, with a higher burden observed in low-income nations [8] and Sub-Saharan African countries [12,13]. Pre-eclampsia has also been linked to maternal and perinatal adversities such as preterm delivery, foetal development retardation, intrauterine foetal death, stillbirth, placental abruption, increased risk for caesarean section delivery, hospitalization, intensive care units admission, low birth weight neonate, small for gestational age neonate, and perinatal death [11,14,15,16,17]. Outcomes from national content-specific studies discovered similar adverse outcomes [18,19,20]. Pre-eclampsia has also been implicated in significant short-term and long-term adversities, including increased susceptibility to a variety of cardiovascular, cerebrovascular, and neurocognitive disorders, as well as diabetes and renal disease for both mother and child post-delivery [21,22,23,24,25]. Short-term consequences of exposure to pre-eclampsia during pregnancy include cerebrovascular haemorrhage, eclampsia, haemolysis, increased liver enzymes, low platelets (HELLP) syndrome, and retinal detachment [11,14,15,16,26]. Posterior reversible encephalopathy syndrome (PRES) during the early postpartum period has been reported in severe cases [27,28,29,30].

Despite the high possibilities of these adverse outcomes occurring with pre-eclamptic pregnancies, the maternal and perinatal outcomes of pregnant women in the Eastern Cape province, particularly in rural settings, have yet to be adequately studied. The Eastern Cape province of South Africa witnessed an increase in maternal mortality in facility ratio (MMFR) per 100,000 live births from 106.1 in 2019 to 108.2 in 2020, higher than the Sustainable Development Goals target of 70 [31]. Hypertensive disorders of pregnancy, which are the family tree of pre-eclampsia, are one of the top three causes of maternal and perinatal death in South Africa [32,33]. In the Maternal Morbidity Measurement framework, hypertensive disorders of pregnancy were the second leading direct cause of maternal morbidity [34] and, when influenced by other conditions [35], contribute significantly to maternal mortality, making research of this nature a priority in guiding maternal health programs and providers. Hence, this research study was designed to ascertain the adverse maternal and foetal outcomes of pre-eclampsia in a health facility that caters to pregnant women from predominantly rural backgrounds. The findings might help mitigate these adverse outcomes and pave the way for clinical scientists to develop interventions to avoid these events.

## 2. Materials and Methods

### 2.1. Study Design

A prospective analytical gravidity matched case-control study was conducted from 5 December 2024 to 5 February 2025 to assess the association between pre-eclampsia and known adverse maternal and foetal outcomes.

### 2.2. Study Setting

The study was conducted at the Labour Ward of Nelson Mandela Academic Hospital (NMAH) in Mthatha. The hospital serves as a tertiary referral hospital for managing complicated pregnancies for inhabitants from historically marginalized districts of the Eastern Cape province. These districts include the OR Tambo district, Chris Hani district, Amatole district, Alfred Nzo district, and Joe Gqabi district, all of which are predominantly rural.

The management of patients with pre-eclampsia at NMAH is guided by the gestational age and the presence of any complications associated with the condition. The primary goal is to ensure that all patients with pre-eclampsia are delivered by 34 weeks and six days of gestation. Patients with pre-eclampsia but without severe features, who are less than 34 weeks and six days pregnant, are typically admitted to the antenatal ward until delivery. Those who develop complications, regardless of their gestational age, are stabilized, and a termination of pregnancy is performed within 8 to 24 h of admission. Complications that warrant termination of pregnancy include imminent eclampsia, eclampsia, HELLP syndrome, placental abruption, foetal distress, preterm labour, pulmonary oedema, and severe hypertension not responding to treatment.

Routine investigations for all pre-eclamptic patients include checks of haemoglobin, platelet count, urea, and electrolytes, aspartate aminotransferase, alanine aminotransferase, and lactate dehydrogenase levels. Additionally, non-stress tests (NSTs) are performed every two to three days, along with ultrasound and Doppler studies every two weeks.

For pharmacological management, antihypertensive medications such as methyldopa and hydralazine are used, along with rapid-acting drugs like nifedipine and labetalol for patients experiencing severely elevated blood pressure (systolic blood pressure ≥ 160 and/or diastolic blood pressure ≥ 110 mmHg). Corticosteroids, specifically betamethasone at a dosage of 12 mg every 12 h (totalling 24 mg), are routinely given to all patients at admission if they are less than 35 weeks pregnant, either at the referring hospital or at NMAH. Magnesium sulphate is administered prophylactically to prevent seizures in cases of severe hypertension or complications such as imminent eclampsia or eclampsia.

Post-delivery, all patients with pre-eclampsia continue to receive magnesium sulphate for at least 24 h. Their blood pressure is carefully managed, and they remain hospitalized for 3 to 5 days or until their condition stabilizes. A follow-up appointment at the postnatal care clinic is scheduled for one week postpartum to monitor blood pressure and assess the need for continued antihypertensive treatment up to 12 weeks after delivery.

### 2.3. Study Population

All the parturients with a diagnosis of pre-eclampsia confirmed by an obstetrician or medical doctor at the NMAH labour ward were considered as cases. The control group was all normotensive parturients at the NMAH labour ward with non-hypertensive disorders of pregnancy.

### 2.4. Inclusion and Exclusion Criteria

Parturients in the labour ward confirmed with pre-eclampsia regardless of parity, gravidity, and age from 13 years based on the youngest maternal age in the hospital database, were included in the study as cases. Normotensive parturients admitted to the labour ward, matched for gravidity with cases at a ratio of 1:1, were included as controls. The controls were parturient with bad obstetric history (previous miscarriages, previous macerated or fresh stillbirth), history of placental abruptio or previa, having placenta previa or abruptio with the index pregnancy, history of or having polyhydramnios or anhydramnios, or high body mass index.

Parturients with chronic hypertension, eclampsia, gestational hypertension, multiple pregnancy, and co-existing heart disease, hepatic disease, and renal disease were not included in the case group. Normotensive parturients with multiple pregnancies, co-existing heart disease, hepatic disease, renal disease, gestational diabetes mellitus, Type 1 or 2 diabetes mellitus, and pregestational hypertension were excluded from the control group. Moreover, normotensive parturients who develop pre-eclampsia within 48 h after delivery were excluded from the control group.

### 2.5. Sample Size Determination

The sample size was estimated using Dupont’s formula for matched case-control studies: $N\;=\;\frac{{\left(OR-1\right)}^{2\;}}{4*(1-p)}\;*\;\frac{{\left(Z\alpha /2\;+\;Z\beta \right)}^{2\;}}{\left[po\;\left(1-po\right)\;+\;p1\;\left(1-p1\right)]*(1\;+\;m\right)}$ [36]. Where: N = Number of matched pairs (cases and controls); Zα/2 is the z-score value for alpha at a 10% significance level for a two-tailed test = 1.645; Zβ is the z-score value for the desired power of 80% = 0.84; Po is the proportion of pre-eclampsia among all pregnant women = 2% (0.02); P1 is the proportion of pre-eclampsia among primigravidae = 10% (0.10); m is the matching ratio of 1 controls per case; p is the correlation coefficient between the exposure status of matched pairs (assumed to be 0.2 since it is unknown); and OR is the odd ratio (effect size) = 2.9. The sample size was 276, rounded up to the nearest ten, giving a sample of 280; 140 cases and 140 controls. The estimated sample size has an enhanced ability to detect real differences since it exceeds 30 participants per group for an 80% power required in a comparative study [37].

### 2.6. Sampling Technique

**Figure 1:**
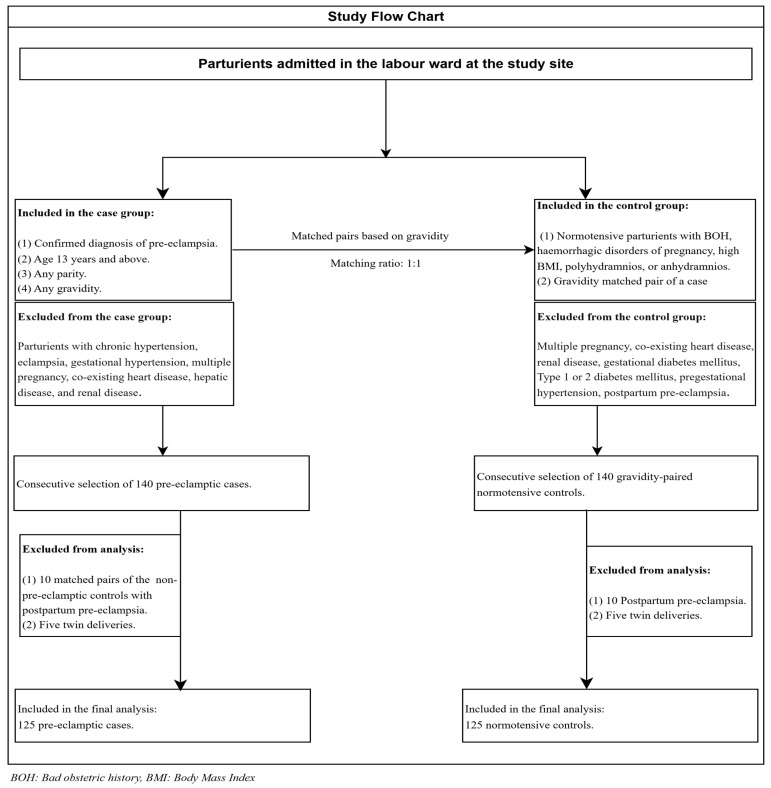
Flow diagram showing study participants.

A total of 280 parturients were recruited: 140 pre-eclamptic cases and 140 normotensive controls. The pre-eclamptic cases were recruited first, and the normotensive controls were matched to the cases based on their gravidity at a ratio of one control to a case. The cases were recruited using a consecutive sampling technique. Likewise, the consecutive sampling technique was used to select matched control pairs. However, based on the inclusion criteria, only 250 were analysed. Ten normotensive controls developed pre-eclampsia after delivery; their paired cases were excluded. Ten had twin deliveries and, as such, were excluded. Therefore, the final analysis included the data obtained from 250 parturients: 125 pre-eclamptic parturients and 125 normotensive parturients (Figure 1).

**Figure 2:**
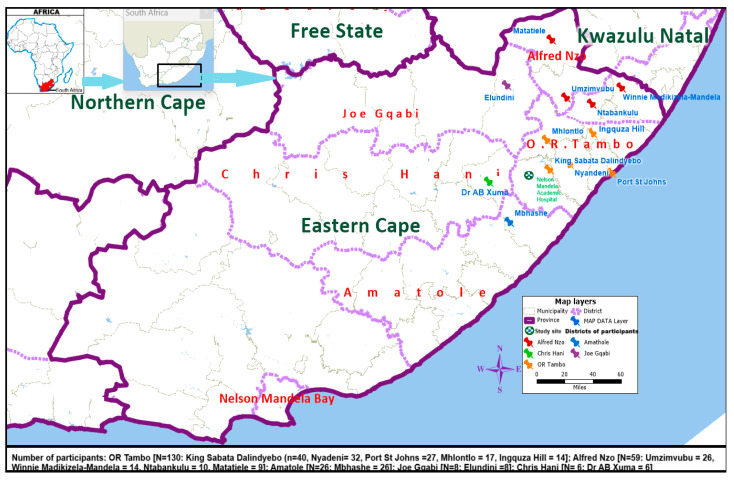
Geographical distribution of the sample population.

Geographically, almost three-quarters (70.4%, n = 176) of the participants reside in villages within 12 local municipalities and 5 district municipalities in the Eastern Cape province of South Africa (Figure 2).

### 2.7. Data Collection

Although the participants were recruited during labour, only data that could be obtained from the files were entered into the questionnaire. All other data were collected after delivery or when the participants’ health was stable. Privacy was ensured during data collection by bed screening or in a private space within the labour unit. Data was collected using a self-designed, pretested, and structured questionnaire adopted from studies conducted within and outside African settings [11,14,15,19]. The questionnaire was available in English and IsiXhosa to obtain sociodemographic, clinical, and obstetrical data. Maternal and foetal outcome variables were obtained from the medical records as documented by the clinicians. The questionnaire was completed within 15 to 20 min during antenatal and postnatal data collection [some text moved to study variables to avoid repetition].

### 2.8. Study Variables

The exposure variable was pre-eclampsia.

The outcome variables were adverse maternal outcomes (for example maternal death, low Glasgow coma scale, HELLP syndrome, extended length of hospital stay, postpartum eclampsia, and specification of other adverse outcomes which were not in the list) and adverse foetal outcomes (for example foetal death, foetal distress, prematurity (delivery before 37 weeks of gestation), low birth weight (birth weight less than 2500 g), low 5 min Apgar score (less than 7), and others).

Sociodemographic variables were place of residence, age, educational level, employment status, marital status, and average monthly income.

Clinical variables include HIV status, time of diagnosis of HIV, antiretroviral drug use, named antiretroviral, and length of hospital stay.

Obstetrical variables include gravidity, parity, antenatal clinic attendance, gestational age at delivery, gestational age at diagnosis of pre-eclampsia, gestational age at delivery, and mode of delivery.

### 2.9. Study Term Definitions

Pre-eclampsia is defined as a new onset of hypertension and either proteinuria or end-organ dysfunction, most often after 20 weeks of gestation in a previously normotensive woman and resolving within 6 weeks after delivery [5]. Any of the four criteria confirms hypertension: (i) if two systolic blood pressure taken four hours apart are higher than or equal to 140 mmHg; (ii) if two diastolic blood pressure taken four hours apart are higher than or equal to 90 mmHg; (iii) if a single systolic blood pressure is higher than or equal to 160 mmHg; or (iv) if a single diastolic blood pressure is higher than or equal to 110 mmHg [5].

Normotensive parturient: a parturient with a maximum systolic blood pressure of less than 130 mmHg, maximum diastolic blood pressure of less than 80 mmHg before 20 weeks of gestation, and no previous diagnosis of chronic hypertension [38].

Gravidity denotes the index and number of previous pregnancies, irrespective of the duration and outcome of the pregnancy [39].

Parity denotes the number of pregnancies reaching 24 weeks and 0 days of gestation or beyond, regardless of the number of foetuses or outcomes. In multiple pregnancies, parity only increases with the birth of the last baby [40].

Gestational age denotes the duration of pregnancy and is estimated using the last menstrual period, symphysis-fundal height, palpation, ultrasound, or a combination of these methods [41].

### 2.10. Reliability and Validity of Data Collection Tool

All the parameters measured and obtained for the study were selected according to the study objectives and from available literature on the topic. The questionnaire was pretested in a pilot study comprising 5% of the sample size (7 pre-eclamptic cases and 7 normotensive controls) to assess the completeness of the representation of required variables, relevance, consistency, clarity, and understanding of the questionnaire. All necessary revisions were made based on the feedback received to improve the quality of the research tool before the actual study initiation.

### 2.11. Statistical Analysis

All collected data were captured on EPI INFO version 7.2.5.0 (Centres for Disease Control and Prevention, Atlanta, GA, USA, 2022) [42], cleaned, and cross-matched before transferring to International Business Machine Statistical Package for the Social Sciences version 29 software (IBM SPSS, Armonk, NY, USA, 2023) [43] for analysis. Continuous variables were described as median, interquartile range (IQR), and when categorized as frequencies and proportions. Bivariate analyses were computed, and a forward Wald binary regression model included the significant bivariate variables to determine pre-eclampsia’s independent adverse outcomes, identified by the variables’ adjusted odds ratios. The model was well-fit based on the Hosmer-Lemeshow significance value of 0.820. A *p*-value of 0.05 at the 95% confidence interval was considered significant.

### 2.12. Ethical Considerations

Ethical clearance was obtained from the Walter Sisulu University Faculty of Medicine and Health Sciences (WSU FMHS) research ethics and scientific review committee (Ref 234/2024), administrative approval was obtained from the Eastern Cape Department of Health, and site permission was granted from the management of the study site. Participants younger than 18 signed the informed consent forms, while those younger than 18 signed the assent forms after permission from the parent or guardian had been established. Participants’ names, personal identification numbers, or other personal identifiers were not gathered, and the questionnaires were assigned coded numbers. The data collection process does not require any harmful procedure; however, care was taken to ensure that the participants were not hurt in any manner, whether emotionally, physically, or otherwise.

## 3. Results

### 3.1. Description of the Sample Population

The data of 250 parturients, with 125 diagnosed with pre-eclampsia, were analysed. The median maternal age was 29 years (IQR: 23 to 34), with the youngest 15 years and the oldest 44 years. The majority were never married (53.6%), not working (74.8%), booked for antenatal care (87.6%), and multigravida (52.8%). Slightly more than a quarter (26.8%) were infected with human immunodeficiency virus (HIV) (Table 1).

**Figure 3:**
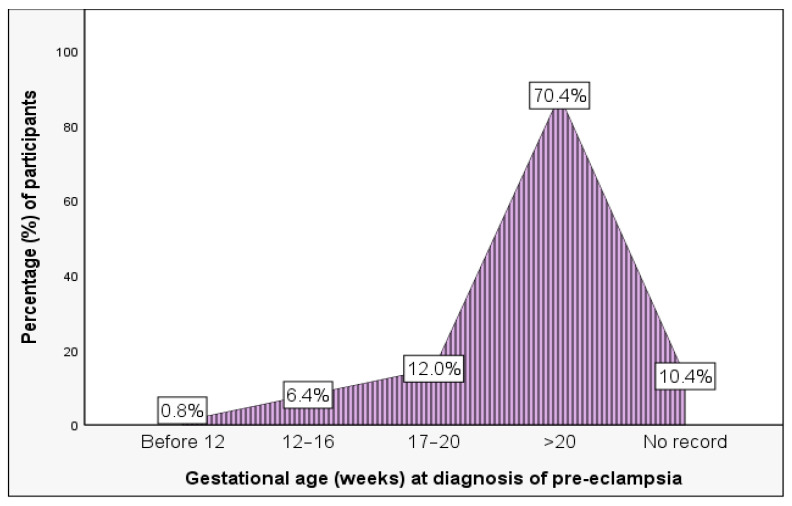
Gestational week at diagnosis of pre-eclampsia.

The median gestational age at diagnosis of pre-eclampsia was 24 weeks (IQR: 21 to 29.5 weeks). Most (70.4%, n = 88) were diagnosed after 20 weeks of gestation (Figure 3).

### 3.2. Adverse Maternal Outcomes

Table 2 presents crude odds ratios (ORs) for the association of adverse maternal outcomes with pre-eclampsia. Preterm delivery (OR = 2.3), caesarean section delivery (OR = 2.4), haemolysis, elevated liver enzymes, low platelets (HELLP) syndrome (OR = 69.8), postpartum eclampsia (OR = 2.1), and more extended hospital stay (OR = 1.2) were significantly associated with pre-eclampsia. Maternal adverse outcomes, including intra-uterine foetal death, low Glasgow coma scale, and hospitalization, were not found to be significantly associated with pre-eclampsia, although they were more frequently observed among the pre-eclamptic cases. There were no maternal deaths during the study period.

Two adverse maternal outcomes were significantly associated with pre-eclampsia in the forward Wald binary regression analysis after adjusting for confounding adverse outcomes. The study revealed HELLP syndrome (OR = 42.7) and preterm delivery (OR = 3.0) as the most dominant adverse maternal outcomes associated with pre-eclampsia (Table 3).

### 3.3. Adverse Foetal Outcomes

Adverse outcomes were more commonly reported among neonates from pre-eclamptic cases. These neonates were significantly more likely to have low birth weights (OR = 2.3), to be premature (OR = 2.3), to experience foetal distress (OR = 2.8), have a low 5 min Apgar score (OR = 2.0), and have absent end diastolic flow (OR = 2.1) based on bivariate analysis. However, neonates from both groups of study participants experienced other adverse outcomes at a statistically similar proportion (*p* > 0.05). The observed rates of adverse outcomes in the pre-eclamptic compared to normotensive control group revealed stillbirth (5.6% vs. 2.4%), foetal growth restriction (2.4% vs. 1.6%); respiratory distress syndrome (2.4% vs. 7.2%); congenital abnormality (0.8% vs. 0.8%); and intensive care unit admission (17.6% vs. 15.2%), with higher rates among neonates of preeclamptic cases except for congenital abnormality, where the rates were equal (Table 4).

The adjusted ORs and 95% confidence intervals are presented in Table 5. Adverse foetal outcomes significantly associated with pre-eclampsia in the multivariate analysis were low birth weight (OR = 2.1) and foetal distress (OR = 2.5).

## 4. Discussion

The study determined the maternal and foetal adverse outcomes associated with pre-eclampsia among pregnant women residing in six mainly rural district municipalities within the Eastern Cape province. The findings highlight the outcomes of pre-eclampsia, a hypertensive disorder of pregnancy considered in the Maternal Morbidity Measurement (MMM) framework as the leading direct cause of maternal morbidity [34,44] and central to the achievement of maternal health and wellbeing, a requirement in Sustainable Development Goal 3 targets 1 and 2 [45,46]. Demographically, the participants were mainly in their twenties and unemployed. Maternal HIV infection (26.8%) was higher than 17.2% among adult South Africans [47] and 19% among pregnant women in the South African Western Cape province [48]. Despite the high rate, a statistically similar proportion of normotensive controls were also HIV infected, refuting any link between HIV and the development of pre-eclampsia as previously documented [49,50], although confirmed elsewhere [51].

Adverse maternal outcomes associated with pre-eclampsia were preterm delivery, HELLP syndrome, caesarean section delivery, and postpartum eclampsia. However, only preterm delivery and HELLP syndrome were definitely due to pre-eclampsia with a 3-fold risk (95% CI: 1.5 to 5.9) and a 42.7-fold risk (95% CI: 5.6 to 323.6), respectively. Preterm delivery occurred in most pre-eclamptic women in the current population, a finding that fits into the available literature [52,53,54]. Preterm delivery risk is reported to be higher for early-onset pre-eclampsia [53,55]. However, in the present study population, preterm delivery was irrespective of the time of diagnosis of pre-eclampsia, suggesting the interplay of other factors such as anaemia, birth spacing of fewer than two years, few antenatal care attendances, and non-usage of supplements [56], thus requiring further analysis to ascertain if any association exists. The findings confirmed a significantly low number of antenatal clinic attendances among women with preterm delivery. However, anaemia, birth spacing, and supplementation were not associated with having preterm delivery. Nonetheless, delivery of the placenta is the commonly applied obstetrical intervention to correct this uteroplacental abnormality [56], and the earlier it occurs, the better. So, the problem here is not preterm delivery but pre-eclampsia itself.

Indeed, at the study site, the management of pregnant women with pre-eclampsia entails delivery by 34 weeks and 6 days of gestation. Thus, there is a need to encourage antenatal clinic attendance and enhance targeted interventions to minimize the risk of developing pre-eclampsia during pregnancy among at-risk women. Other interventions should also be considered, including early identification of pre-eclampsia for prompt management and prevention—for example, calculating the risk of pre-eclampsia using predictive risk models [57] and biomarkers [3,58,59,60]. In terms of biomarkers, a low ratio of soluble FMS-like tyrosin kinase-1 (sFlt-1) and placental growth factor (PlGF) rules out the possibility of a pregnant woman developing pre-eclampsia, while a high ratio indicates the potential of its occurrence [61]. Other useful biomarkers include reduced plasma protein-A [3,58] and low placental protein-13 [59,60].

The association of HELLP syndrome with pre-eclampsia has been confirmed in recent studies [62,63,64]. The pathway for the development of HELLP syndrome follows the path laid by pre-eclampsia. This pathway has been explained by the dysfunctional placenta, impaired placenta, placental ischaemia, endothelial cell dysfunction, and placental antiangiogenic factors, which activate liver sinusoidal endothelial cells [3,65,66,67,68]. A recent study found that pregnant women with pre-eclampsia had prolonged median prothrombin time (PT), prolonged activated partial thromboplastin time (aPTT), high mean platelet volume, high Platelet Distribution Width (PDW), and low mean platelet count [69]. Since HELLP syndrome alters serum components, including red blood cells and platelets, routine laboratory tests of coagulation and platelet parameters for pregnant women with pre-eclampsia should be applied to reduce the risk of coagulation and platelet abnormalities [69]. Fortunately, some of these investigations are routinely carried out at the study site. Although the time of diagnosis of HELLP syndrome among the participants is uncertain, the occurrence contributed to preterm delivery, as it is one of the indications for immediate delivery at the study site.

The fact that caesarean section delivery was not performed solely among pre-eclamptic women in the present study could be because it is a life-saving procedure and can be opted for by normotensive women presenting with risky conditions. However, the Robson classification was not examined to support the findings. Additionally, postpartum eclampsia, a documented nervous system complication [70,71], did not show as an independent adversity of pre-eclampsia. The determinants of eclampsia in pre-eclamptic women are younger age (14 to 19 years) and primiparity [72]. The present study’s findings confirm no significant association between postpartum eclampsia and these determinants, which could probably be why it is unrelated to pre-eclampsia. Although postpartum eclampsia occurs only among the pre-eclamptic cases, most of them were older than 20 years and were either nulliparous or multiparous.

Adverse foetal outcomes due to pre-eclampsia identified were low birth weight, foetal distress, prematurity, low 5 min Apgar score, and absent end diastolic flow, with the last three showing no significant associations. The significant association between low birth weight and pre-eclampsia is consistent with findings from a study conducted in China [73], where low birth weight was an associated adverse perinatal outcome of pre-eclampsia. The determinant for having a low birth weight neonate was if pre-eclampsia was of early onset [73]. This could be the reason in the present study, as the median gestational week was 20, and almost a quarter (21.4%) were diagnosed with pre-eclampsia before the 20th week of gestation. Early onset of pre-eclampsia is rare but occurs, as reported in a recent systematic review [4]. Furthermore, a positive correlation was observed between birth weight and gestational week at diagnosis in the present study, but the relationship was very weak and insignificant (*p* = 0.144, correlation coefficient = 0.139). This finding requires an in-depth understanding of factors contributing to low birth weight among pre-eclamptic cases, as low birth weight is the primary contributor to perinatal death in sub-Saharan African countries [74].

Likewise, women whose foetus was exposed to distress were 4 times (CI: 2 to 7.8 times; *p* < 0.001) more likely to be pre-eclamptic. Previous studies mainly reviewed pre-eclampsia as associated with foetal distress [1,75]. A survey carried out in China also found an increased incidence of foetal distress [63]. Foetal distress in pre-eclamptic pregnancy could be due to aberrant placental implantation [65,66], which can cause inadequate uterine and placental perfusion, and consequently hypoxia and elevated oxidative stress [65].

Prematurity was not associated with pre-eclampsia in the present study, inconsistent with the national [19] and international [11,14,16] literature. The relative risk for this occurrence becomes stronger with early onset (<28 weeks) pre-eclampsia [55]. This inconsistent finding could be due to the interaction between the variables in the regression model, which covered the association, as this variable was identified as a significant adverse maternal outcome with pre-eclampsia. Indeed, the exclusion of low birth weight in the model presented prematurity as an adverse outcome, and the combination of the two presented only low birth weight as an adverse outcome. This implies that most of the premature babies had low birth weight, which was confirmed in the present findings as 77.8% of premature babies had low birth weight and only 22.2% had normal weights, with a crude odds ratio of 11 (95% CI 6.1 to 19.7; *p* < 0.001). Furthermore, the present study revealed a strong positive correlation (correlation coefficient 0.8, *p* < 0.001) between birth weight (kg) and gestational age at delivery (weeks).

A low Apgar score also showed no significant association with pre-eclampsia in the present study, despite the increased risk of occurrence reported in a recent Swedish study [76]. This association is commonly observed among women from rural areas who deliver before 30.5 weeks of gestation, who developed pre-eclampsia before 28.5 weeks of gestation, and or who had a caesarean delivery [77,78]. The present study observed a moderate positive significant correlation between the 5 min Apgar score and gestational age at delivery (r = 0.5, *p* < 0.001). An extremely weak positive correlation existed between the 5 min Apgar score and gestational age at diagnosis (correlation coefficient = 0.01, *p* = 0.948). Moreover, 73.4% of neonates delivered through caesarean section had normal 5 min Apgar scores in the present study. The low rate of occurrence of these determinants of low Apgar score could be the probable reason for its exclusion as an adverse outcome of pre-eclampsia.

Again, absent end diastolic flow showed no significant occurrence because of pre-eclampsia, although it was identified as a bivariate associate factor, occurring only in pre-eclamptic cases. Absent end-diastolic flow was reported as one of the abnormal blood flows in the umbilical artery disorders among pre-eclamptic pregnant women [79]. The umbilical arterial disorder has been pointed out as the cause of foetal growth retardation [79,80]. While true, no such association could be pointed out in the current population. All the cases with foetal growth retardation were not accompanied by absent end-diastolic flow.

The strongest aspect of this study was that it was conducted with a scientifically sufficient number of patients. It was the first study in which adverse maternal and foetal outcomes of pre-eclampsia were examined in the rural Eastern Cape. Moreover, the study setting provides antenatal care services to more than 90% of pregnant women with pre-eclampsia as referral from lower levels of health care in the district is made immediately after a diagnosis of pre-eclampsia is established, confirming the unlikely influence of the three delay models [35] in outcomes and supported by the zero maternal mortality. Strengthened by these features, adverse maternal and foetal outcomes were identified. The matched case control study employed accurately shows the association between pre-eclampsia and these adverse maternal and foetal outcomes. Still, it cannot be concluded that these adverse outcomes were caused by pre-eclampsia. Nonetheless, since the study design is intended to identify associated outcomes, it is crucial to provide high-quality antenatal care for early identification of pregnant women with pre-eclampsia, pregnant women at risk of developing pre-eclampsia, and to minimize these adverse outcomes.

Despite these contributions, the study was limited because the data were collected using a tool that had not been used before and with no known reliability coefficient, as there is no publicly available tool for assessing the intended outcomes. Cognizant of this, care was taken to ensure that the measured variables included known maternal and foetal outcomes of pre-eclampsia. The validated model, such as the fullPIERS model, could not be used as it is designed to stratify pregnant women at risk of adverse maternal outcomes within 48 h and seven days of diagnosis of pre-eclampsia. Meanwhile, the present study aimed to determine the actual adverse maternal and foetal outcomes for which no validated tool is available. The research tool used was pretested in a pilot study and revised before the initiation of the study.

Future research should consider conducting a retrospective analysis to confirm the risk of adverse maternal outcomes using the fullPIERS risk prediction model and the actual outcomes at term and post-delivery. Future research should also focus on profiling obstetrical complications to inform the maternal morbidity framework specific to rural South African settings. Lastly, future studies should delve into biomarkers and genetic markers of the different obstetrical complications and investigate how to incorporate such findings into antenatal care services.

## 5. Conclusions

This study shares baseline knowledge and opens the floor for a debate on adverse maternal and foetal outcomes of pre-eclampsia, from a tertiary hospital serving mainly rural, economically disadvantaged pregnant women. The information shared could help with possibly enhancing the monitoring of pre-eclamptic women and improving maternal and foetal care to prevent the identified adverse outcomes. Health care providers should therefore focus on sensitizing the population to the need for early antenatal visits, continuous monitoring of pre-eclamptic pregnant women, and implementation of preventive and curative measures to reduce the possibilities of this condition and its adverse outcomes. Specifically, policies should stipulate the need to screen at-risk pregnancies using biomarkers for early identification of pre-eclampsia for early management and identification of pre-eclamptic pregnant women at risk of adverse outcomes at the time of diagnosis using risk predictive calculators. Clinicians should strengthen the implementation of tested good practice care plans for women at risk of preterm birth and HELLP syndrome by administering antenatal corticosteroids to improve newborn outcomes, administering magnesium sulphate for foetal protection against neurological complications, administering progestogen or performing cervical cerclage, or inserting a pessary to extend gestational length. Lastly, public health practitioners should consider these findings in formulating health education programs on risk factors of pre-eclampsia, management options, and consequences of pre-eclampsia to both mother and child dyads.

## Figures and Tables

**Table 1:** Demographic and obstetrical characteristics of 250 pre-eclamptic cases and normotensive controls.

Variables of Interest	Pre-Eclamptic Cases (N = 125); n (%)	Normotensive Controls (N = 125); n (%)	All (n = 250); n (%)	*p*-Value
Age Group (years)				
<20 years	25 (20.0)	13 (10.4)	38 (15.2)	0.064
20–34 years	69 (55.2)	84 (67.2)	153 (61.2)	
≥35 years	31 (24.8)	28 (22.4)	59 (23.6)	
Age (years): Median (IQR)	28 (21–34)	29 (24–34)	29 (23–34)	0.483
Marital Status				
Never married	72 (57.6)	62 (49.6)	134 (53.6)	0.434
Married	15 (12.0)	19 (15.2)	34 (13.6)	
Not stated	38 (30.4)	44 (35.2)	82 (32.8)	
Educational Level				
Below Grade 12	44 (35.2)	39 (31.2)	83 (33.2)	0.234
Certificate	42 (33.6)	43 (34.4)	85 (34.0)	
Degree and above	21 (16.8)	32 (25.6)	53 (21.2)	
No response	18 (14.4)	11 (8.8)	29 (11.6)	
Employment				
Unemployed	91 (72.8)	96 (76.8)	187 (74.8)	0.254
Employed	27 (21.6)	27 (21.6)	54 (21.6)	
No response	7 (5.6)	2 (1.6)	9 (3.6)	
Average Monthly Income				
ZAR < 2000.00	72 (59.5)	75 (61.5)	147 (60.5)	0.256
ZAR 2000.00–R 5000.00	29 (24.0)	26 (21.3)	55 (22.6)	
R > 5000.00	13 (10.7)	19 (15.6)	32 (13.2)	
No response	7 (5.8)	2 (1.6)	9 (3.7)	
HIV Status				
Positive	32 (25.6)	35 (28.0)	67 (26.8)	0.645
Negative	86 (68.8)	86 (68.8)	172 (68.8)	
Unknown	7 (5.6)	4 (3.2)	11 (4.4)	
Time of Diagnosis of HIV				
Before pregnancy	21 (53.8)	29 (74.4)	50 (64.1)	0.108
During pregnancy	9 (23.1)	3 (7.7)	12 (15.4)	
Unknown	9 (23.1)	7 (17.9)	16 (20.5)	
Gravidity Group				
Primigravida	40 (32.0)	40 (32.0)	80 (32.0)	1.000
Multigravida	66 (52.8)	66 (52.8)	132 (52.8)	
Grand Multigravida	19 (15.2)	19 (15.2)	38 (15.2)	
Gravidity	2 (1–4)	2 (1–4)	2 (1–4)	1.000
Antenatal clinic booking				
Booked	112 (89.6)	107 (85.6)	219 (87.6)	0.443
Unbooked	13 (10.4)	18 (14.4)	31 (12.4)	
Number of Antenatal Clinic Visits Attended	3 (1–4)	2 (2–4)	2 (1–4)	0.193

IQR: Interquartile range; HIV: Human immunodeficiency virus.

**Table 2:** Adverse maternal outcomes observed in a sample of 250 pre-eclamptic cases and normotensive controls.

Maternal Outcome	Pre-Eclamptic Cases (N = 125); n (%)	Normotensive Controls (N = 125); n (%)	Crude Odd Ratio	95% Confidence Interval
Preterm Delivery	76 (60.8)	50 (40.0)	2.3 *	1.4–3.9
Caesarean delivery	115 (92.0)	103 (82.4)	2.4 *	1.1–5.4
Intra-uterine Foetal Death	3 (2.4)	0 (0.0)	2.0	1.8–2.3
HELLP Syndrome	45 (36.0)	1 (0.8)	69.8 *	9.4–516.1
Postpartum Eclampsia	10 (8.0)	0 (0.0)	2.1 *	1.8–2.4
Low Glasgow Coma Scale	2 (1.6)	0 (0.0)	2.0	1.8–2.3
Hospital Admission	101 (80.8)	99 (79.2)	1.1	0.6–2.1
Days of Hospital Admission	6 (5–9)	5 (4–7)	1.2 *	1.0–1.3

* A *p*-value of less than 0.05 is considered to be statistically significant. HELLP: haemolysis, Elevated liver enzymes, and low platelets.

**Table 3:** Independent adverse maternal outcomes associated with pre-eclampsia.

Adverse Maternal Outcome	Adjusted Odds Ratio	95% Confidence Interval	*p*-Value
Step 1			
HELLP Syndrome	54.6	5.6–323.6	<0.0001
Step 2			
Preterm Delivery	3.0	1.6–5.9	0.001
HELLP Syndrome	42.7	5.6–323.6	<0.0001

**Table 4:** Adverse neonatal outcomes observed in a sample of 250 pre-eclamptic cases and normotensive controls.

Adverse Neonatal Outcomes	Pre-Eclamptic Cases (N = 125); n (%)	Normotensive Controls (N = 125); n (%)	Crude Odd Ratio	95% Confidence Interval
Birth weight categories				
Low birth weight	77 (61.6)	51 (40.8)	2.3 *	1.4–3.9
Normal birth weight	48 (38.4)	74 (59.2)		
Outcome at birth				
Alive	118 (94.4)	122 (97.6)	0.4	0.1–1.6
Stillbirth	7 (5.6)	3 (2.4)		
Apgar score at 5 min				
Low	41 (32.8)	25 (20.0)	2.0 *	1.1–3.5
Normal	84 (67.2)	100 (80.0)		
Premature	76 (60.8)	50 (40.0)	2.3 *	1.4–3.9
Oligohydramnios	3 (2.4)	0 (0.0)	2.0	1.8–2.3
Foetal distress	57 (45.6)	29 (23.2)	2.8 *	1.6–4.8
Foetal growth restriction	3 (2.4)	2 (1.6)	1.5	0.2–9.2
Respiratory distress syndrome	3 (2.4)	9 (7.2)	0.3	0.1–1.2
Absent end-diastolic flow	6 (4.8)	0 (0.0)	2.1 *	1.8–2.3
Congenital abnormalities	1 (0.8)	1 (0.8)	1.0	0.1–16.2
Admission to the intensive care unit	22 (17.6)	19 (15.2)	1.2	0.6–2.3

* A *p*-value of less than 0.05 is considered to be statistically significant.

**Table 5:** Adverse neonatal outcomes observed in a sample of 250 pre-eclamptic cases and normotensive controls.

Adverse Neonatal Outcomes	Adjusted OR	95% Confidence Interval	*p*-Value
Step 1			
Foetal distress	2.8	1.6–4.8	<0.0001
Step 2			
Low birth weight	2.1	1.2–3.5	0.006
Foetal distress	2.5	1.4–4.4	0.001
Step 3			
Low birth weight	2.1	1.2–3.5	0.006
Foetal distress	2.5	1.4–4.4	0.001
Absent end-diastolic flow	>100	(0.0–0.0)	0.999

## Data Availability

Data supporting reported results will be made available upon request.

## Abbreviations

The following abbreviations are used in this manuscript:

MMFR	Maternal mortality in facility ratio
ACOG	American College of Obstetricians and Gynaecologists
WHO	World Health Organisation
HELLP	Haemolysis, increased liver enzymes, low platelets
HIV	Human immunodeficiency virus
